# On impact of transport conditions on variability of the seasonal pollen index

**DOI:** 10.1007/s10453-016-9459-x

**Published:** 2016-10-24

**Authors:** M. Sofiev

**Affiliations:** Finnish Meteorological Institute, Erik Palmenin Aukio, 1, Helsinki, Finland

**Keywords:** Seasonal pollen index, Pollen load, Atmospheric transport, Pollen inter-annual variability, Pollen dispersion modelling

## Abstract

This discussion paper reveals the contribution of pollen transport conditions to the inter-annual variability of the seasonal pollen index (SPI). This contribution is quantified as a sensitivity of the pollen model predictions to meteorological variability and is shown to be a noticeable addition to the SPI variability caused by plant reproduction cycles. A specially designed SILAM model re-analysis of pollen seasons 1980–2014 was performed, resulting in the 35 years of the SPI predictions over Europe, which was used to compute the SPI inter-annual variability. The current paper presents the results for birch and grass. Throughout the re-analysis, the source term formulations and habitation maps were kept constant, which allowed attributing the obtained variability exclusively to the pollen release and transport conditions during the flowering seasons. It is shown that the effect is substantial: it amounts to 10–20% (grass) and 20–40% (birch) of the observed SPI year-to-year changes reported in the literature. The phenomenon has well-pronounced spatial- and species-specific patterns. The findings were compared with observation-based statistical models for the SPI prediction, showing that such models highlight the same processes as the analysis with the SILAM model.

## Introduction

Seasonal pollen index (SPI) is a frequently used quantitative measure of severity of pollen seasons and a proxy for allergenic exposure of the population (D'Amato et al. 1998; D’Amato et al. 2007; Gioulekas et al. 2004). Mechanisms determining it for each particular taxon differ (Dahl et al.: Chapter 3 in Sofiev and Bergmann 2013 and references therein), but several characteristic features can be highlighted.

For trees and many other perennial plants, the reproductive effort varies between the years and the flowering of a year 1 is affected by the environmental conditions and the flowering intensity during the previous year 0 (Dahl and Strandhede 1996; Galán et al. 2001; Latalova et al. 2002; Ranta et al. 2008; Rodríguez-Rajo et al. 2006; Stach et al. 2008). A number of hypotheses have been suggested to explain the apparent systematic patterns in the succession of years with high and low SPI, as well as a strong regional synchronisation of the flowering intensity (Dahl et al.: Chapter 3 in Sofiev and Bergmann 2013 and references therein). Certain success of the SPI-predicting statistical models based on the year-0 pollen observations and the weather conditions confirms the concept. However, the efficiency of these models is often far from being perfect and is limited to (sub-) regional scale with just a few aerobiological stations involved. It was also shown that correlation between the number of catkins for birch and the SPI in Northern Europe is as low as 0.52 (Ranta et al. 2008), i.e. for birch, the regional pollen production (assumed proportional to the number of catkins) has quite limited explanatory and predictive capacity for the SPI in the same region. Other factors have to be involved.

Numerous studies demonstrated that pollen grains are subject to regional- and large-scale atmospheric transport. It is particularly important for wind-pollinating trees due to small size of the grains and substantial release height (Belmonte et al. 2000; Corden et al. 2002; Damialis and Gioulekas 2005; Hjelmroos 1992; Latalova et al. 2002; Mahura et al. 2007; Ranta and Satri 2007; Ranta et al. 2011; Rantio-Lehtimaki 1994; Siljamo et al. 2008; Skjøth et al. 2008; Sofiev et al. 2006, 2012a; Yli-Panula et al. 2009), see also reviews of Smith et al. (2014), Sofiev and Bergmann (2013). Importance of the transport is well recognised for pollen forecasts during the season when the atmospheric dispersion conditions play decisive role in the formation of daily pollen concentration pattern (Siljamo et al. 2006, 2008; Sofiev et al. 2006, 2015a, b; Veriankaitė et al. 2010; Zink et al. 2012, 2013). Atmospheric transport was also mentioned as a potentially significant factor affecting the SPI (Ranta et al. 2008; Sofiev and Bergmann 2013, Chapters 3 and 5), but no quantitative assessments were elaborated.

For grasses, the situation is different: (1) larger pollen grains and low release height suggest lower importance of the atmospheric large-scale transport, (2) herbaceous plants production is more affected by the year-1 pre-season weather conditions than by year-0 situation—at least the bulk of grass models suggests so (Emberlin et al. 1993; Smith et al. 2009; Jones 1995 as cited in Sofiev and Bergmann 2013, Chapter 3). For such plants, the atmospheric conditions over the emission area are more important than characteristics of the large-scale atmospheric transport.

For both tree and herbaceous pollen, the impact of atmospheric transport on daily pollen concentrations is bound to be higher than the impact on the SPI. Firstly, the bulk of the released pollen is deposited in the near vicinity of the sources (Raynor et al. 1970; Tampieri et al. 1977; Wright 1952, 1953), i.e. the large-scale transport affects only a minority of the emitted pollens. Secondly, integration of the concentrations over the season averages out the impact of individual episodes. As a result, the influence of the transport conditions on the SPI is usually assumed to be negligible, and very few studies so far analysed it.

Among the few studies considering the impact of the year-1 conditions on the SPI, Latalova et al. (2002) and Stach et al. (2008) constructed statistical models for birch SPI in Poland and the UK. Apart from that, a qualitative analysis for *Alnus* and *Corylus* in the UK was conducted by Emberlin et al. (2007). They showed that statistical models based on a combination of the past- and present-year meteorology and the past-year SPI give better results than the models based only on past-year meteorology and SPI. Finally, 7-year-long SILAM model simulations for ragweed demonstrated that about half of both the inter-annual variability and the upward trend in Hungary can be attributed solely to transport conditions during flowering (Prank et al. 2013).

A related analysis of the modelled 30-year trends of the SPI and their relations to some meteorological factors is presented in the review of Sofiev and Prank (2016). It attempts to identify the relations between the mean meteorological characteristics over Spain, Germany, and Finland (wind speed, rain intensity and frequency, temperature, etc.) with the modelled SPI. The revealed correlations between the meteorological and multi-decade SPI trends appeared quite limited, suggesting shorter time scale of the possible inter-connections. Therefore, the inter-annual variability seems to be a good target for the analysis.

The current paper uses the results of a 35-year-long SILAM re-analysis that includes many species of anthropogenic and natural origin, in particular six pollen types. For the purposes of the current paper, birch and grass have been selected. The goal of the paper is to quantify the impact of the atmospheric transport conditions on the seasonal pollen index of these two species.

The paper is organised as follows: The next Sect. 2 presents the concept of the study and the SILAM pollen model. Section 3, Results, presents its main outcome, which is discussed in Sect. 4.

## Methodology and input data

### Working hypothesis and concept of the study

The working hypothesis of the study is that the SPI variability from year to year is determined by a combination of three sets of factors:

1. Biological reproductive cycles, primarily controlled by the flowering history and meteorology of the previous year 0 (perennial plants) and pre-season conditions of the current year 1 (primarily, annual plants). These natural biological processes modify the amount of pollen produced by the plant and released during the season of the year 1.
2. Variation of the habitation areas of the plants and their management. This is an external forcing of mainly anthropogenic origin, which can sharply modify the abundance of the plant in a region or reduce its pollination (e.g. mowing of grass during its flowering).
3. Meteorological conditions of the year 1 during the flowering period. They control the dispersion of the actually released pollen and largely decide the near-surface concentrations both in the vicinity of the sources and in the remote areas.

The current study addresses the third set of factors—meteorological conditions of the year 1—and aims at their separation from the biological cycles and anthropogenic forcing.

Observations register cumulative effect of all these factors, and their separation is not straightforward. Dispersion modelling, to the contrary, provides the necessary instrument: one can run the model through many years with “frozen” pollen source terms. Such artificial fixation of the pollen source in the model eliminates the impact of biological cycles and anthropogenic forcing: in each year, the model releases the same total amount of pollen as during all other seasons. Concentrations and, consequently, modelled SPI are affected by the atmospheric transport, which becomes the only source of the modelled SPI variability between the years. In essence, the effect is quantified via sensitivity of the SILAM seasonal pollen predictions to meteorological conditions of the specific year.

It should be kept in mind that fixing the emission parameters and disregarding the above factors (1) and (2) make the “modelled SPI” per se an artificial parameter: it is not supposed to reproduce the actually “observed SPI” for each year. However, the construction and calibration of the SILAM pollen source term ensure that the multi-annual average of the modelled SPI meets the observed values (Sofiev et al. 2015a).

### SILAM model

Implementation of the above concept is based on the System for Integrated modeLling of Atmospheric coMposition (SILAM, http://silam.fmi.fi). The SILAM pollen emission module presently covers 6 taxa: birch, grass, olive, ragweed, alder, and mugwort. The current paper discusses the variability of SPI for birch and grass.

#### Pollen productivity maps for birch and grass

**Fig. 1:**
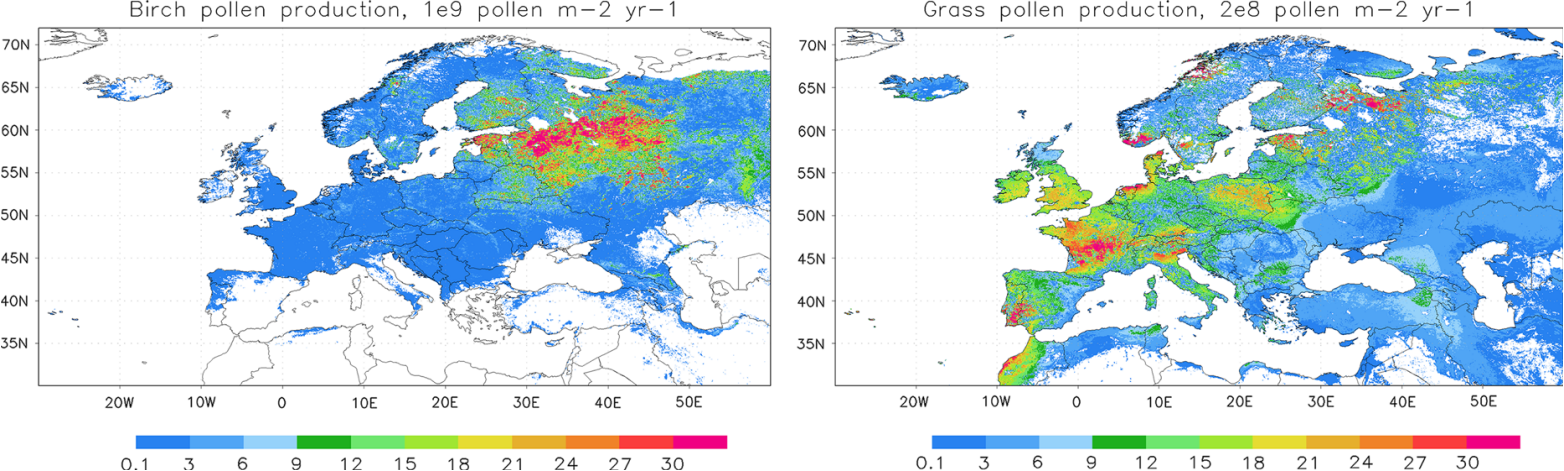
Birch (*left*) and grass (*right*) pollen production maps. Units: birch (10^9^ pollen m^−2^ year^−1^), grass (2 10^8^ pollen m^−2^ year^−1^)

Pollen emission in the current SILAM v.5.4 is based on pollen production maps (Fig. 1, left panel for birch and right panel for grass). They cover the whole Europe and nearby regions and have the resolution of 1 km (birch) and 2 km (grass), owing to the underlying habitation maps of European Forest Institute (Schuck et al. 2000) combined with the global ECOCLIMAP dataset (Masson et al. 2003). Calibration of the pollen production followed the inverse problem technique of Vira and Sofiev (2012), aiming at in average unbiased representation of pollen concentrations observed by the European Aeroallergen Network EAN—see Prank et al. (2013) for details of the procedure. The ideology has some similarity with Skjøth et al. (2010) used for ambrosia inventories by Karrer et al. (2015), Thibaudon et al. (2014), but a principal difference is that the transport of pollen is explicitly included through the SILAM-based data assimilation. As a result, the pollen production maps as shown in Fig. 1 ensure unbiased representation of the multi-annual mean SPI.

#### Birch source term

The birch pollen emission model and its evaluation against the European Aeroallergen Network (EAN) data are described in Sofiev et al. (2012b), Siljamo et al. (2012), whereas its application in the multi-model MACC ensemble, additional evaluation for two specific years and analysis of the SPI representation can be found in Sofiev et al. (2015a).

The pollen presentation model follows the concept of the heat-sum accumulation as the main determinant of the flowering start and end, with the double-heat-sum approach of Linkosalo et al. (2010) applied during the flowering period. The presented pollen is stored in a buffer for ready-to-fly grains waiting for the favourable release conditions, which depend on short-term meteorological developments modulating, promoting, and inhibiting the pollen release (Sofiev et al. 2012b; Siljamo et al. 2012). In more detail, buffered approach is described in Prank et al. (2013) and Zink et al. (2012, 2013).

The pollen vertical release profile is homogeneous starting from 1 m up to 50 m height above the ground. The upper boundary reflects the pollen up-mixing during the discrete emission time step (10 min in the current study).

#### Grass source term

**Fig. 2:**
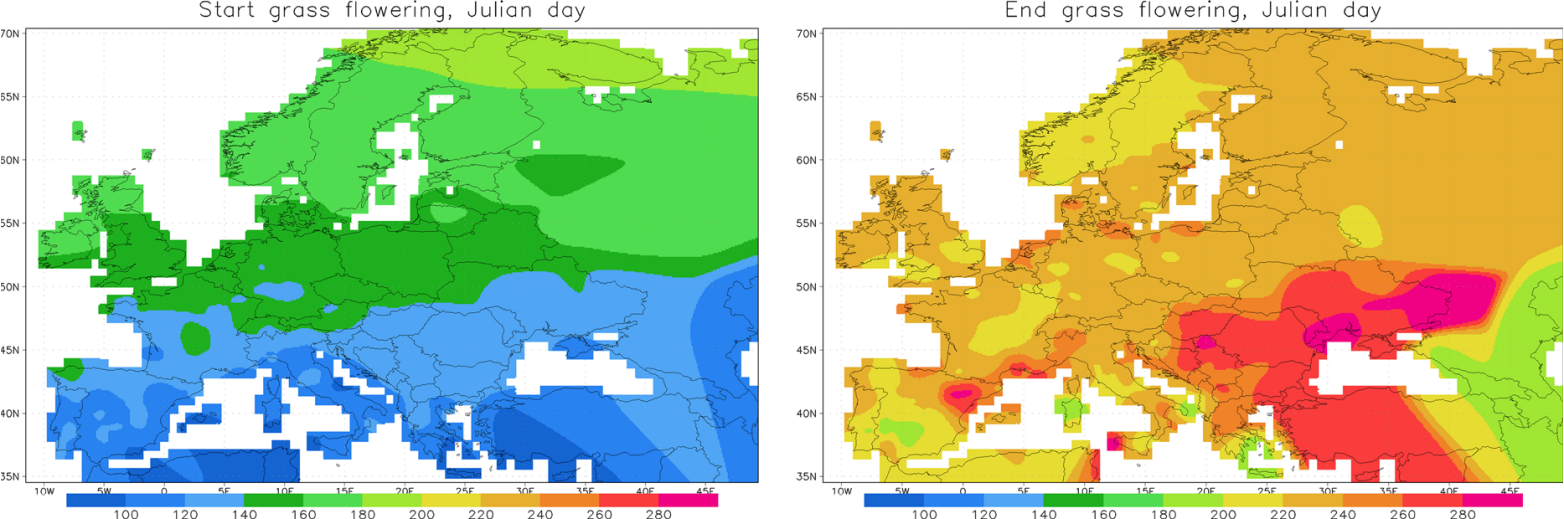
Start and end dates of the grass pollen emission season. Unit: (Julian day since the start of the year)

The grass emission model is the simplest one in the SILAM pool and represents a climatological description of the pollen emission outlined by Sofiev et al. (2006). Unlike the birch source, the grass emission computations do not rely on heat sum. The season starts and ends at prescribed calendar days, specific for each location (Fig. 2). This approach generally follows the other climatology-relying works, such as Bolmgren et al. (2012), Rotzer and Chmielewski (2001), or Ribeiro et al. (2007), but does not involve parameterisations of the spatial patterns, instead storing the mean dates as maps and using interpolation between the reference points.

One of the reasons for the simplified approach is that there are over 200 different grass species in Europe, which pollen cannot be distinguished in the microscopic analysis and which habitation cannot be singled-out from the satellite-originated grassland maps. Therefore, SILAM considers “general grass”, which is a mixture of all those species.

**Fig. 3:**
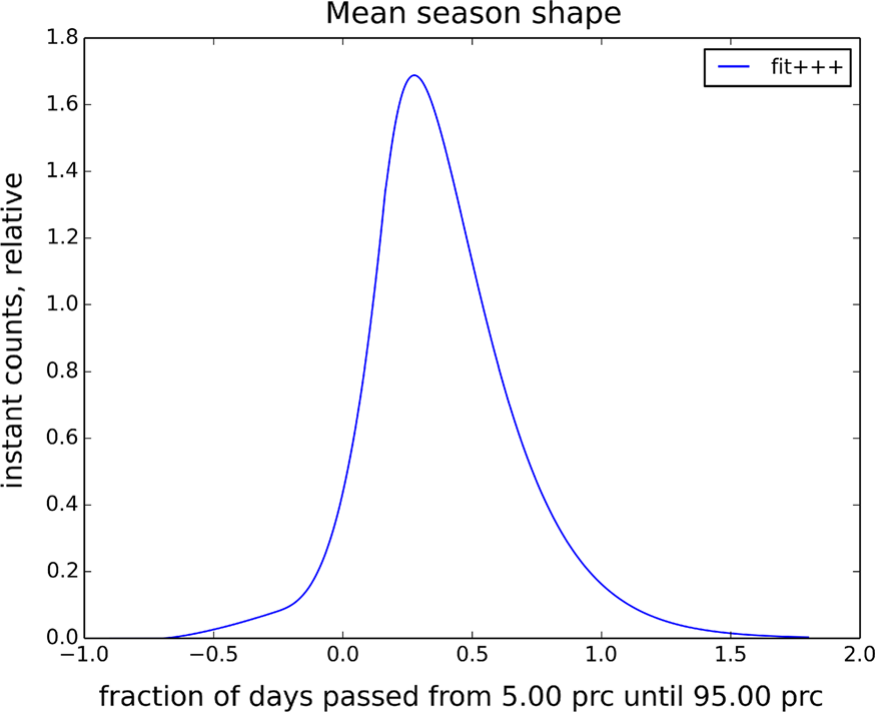
Prescribed shape of grass flowering intensity. Start of the season (*t* = 0) corresponds to the *left panel* of Fig. 2, end (*t* = 1)—to the *right panel*. Emission starts before *t* = 0 and ends after *t* = 1 to meet the aerobiological season start–end definition as 5–95% of the SPI, respectively (Jato et al. 2006)

From the start until the end of flowering, the grass pollen presentation rate is assumed proportional to the season shape curve (Fig. 3). In each grid cell, this curve is multiplied with grass productivity (Fig. 1) and stretched from the season start until the end (Fig. 2).

The release from the ready-to-fly buffer is controlled by the same dynamic flowering-modulating processes as for the birch model: pollen release is suspended during rain and high-humidity periods but strengthened by wind and turbulence (Sofiev et al. 2012b).

#### Input meteorological data

Input meteorological data for the re-analysis were taken from the European Re-Analysis ERA-Interim, which covered the globe for the period 1980–2014. The resolution of the meteorological fields was 0.72 degrees, i.e. about 40 km × 80 km lon–lat at 50N. Due to varying amount of assimilated meteorological information in different years, ERA-Interim is not a homogeneous dataset, especially what refers to the middle and upper atmosphere (Dee et al. 2011). However, near-surface variables in regions with long history of abundant atmospheric observations, such as Europe, are quite stable: the amount of assimilated observations is sufficient for constraining the meteorological model already in 1980s.

### Set-up of the study

Fixation of the source term (Sect. 2.1) has to be carefully carved: flowering progress and pollen release depend on actual meteorology during the season of the year 1 and thus should be accounted for. What should be kept frozen is the parameterisation of such dependence: model formulations must stay frozen through the whole experiment. Secondly, the total number of pollen grains released during each season should be the same for all years. For trees, it is the very number that decided by the year-0 conditions. For grasses, both the habitation map and emission parameterisation were kept the same for all years.

It was expected that the tree pollen has a substantial transport-induced SPI variability. For grasses, the effect is presumably smaller than for birch due to limited impact of transport on these large pollens emitted near the surface.

In SILAM, pollen emission is controlled by: (1) high humidity that slows it down and suspends when RH > 80%, (2) rain that stops the release, and (3) wind that promotes it (Sofiev et al. 2012b; Siljamo et al. 2012). However, none of these processes affect the total amount of pollen released to the air during the whole season: once suspended by, for example, rain, emission resumes when conditions improve. From the SPI standpoint, these are rather the mechanisms that increase it: pollen is not released in rainy periods, which prevents it from being scavenged out. Wind has a dual impact: it promotes the release and allows pollen to be distributed wider, i.e. the near-source area gets less pollen, whereas distant areas receive the extra load. Importantly, strong winds are frequently near atmospheric frontal systems and also associated with rains. Therefore, the combination of humid/rainy periods correlated with wind is bound to affect the SPI in a complicated manner.

The set-up of the runs was the same for all species. The model was run from 1980 till 2014, covering Europe with a grid spanning from (11.5W, 34.5N) till (49.5E, 70.5N) with spatial resolution of 0.1°×0.1° lon–lat (about 5 km ×10 km). Vertical consisted of 8 stacked layers up to 4.8 km above the ground, the lowest layer being 25 m thick. Internal model time step was 10 min, and the output step was 1 h.

## Outcome of the computations

**Fig. 4:**
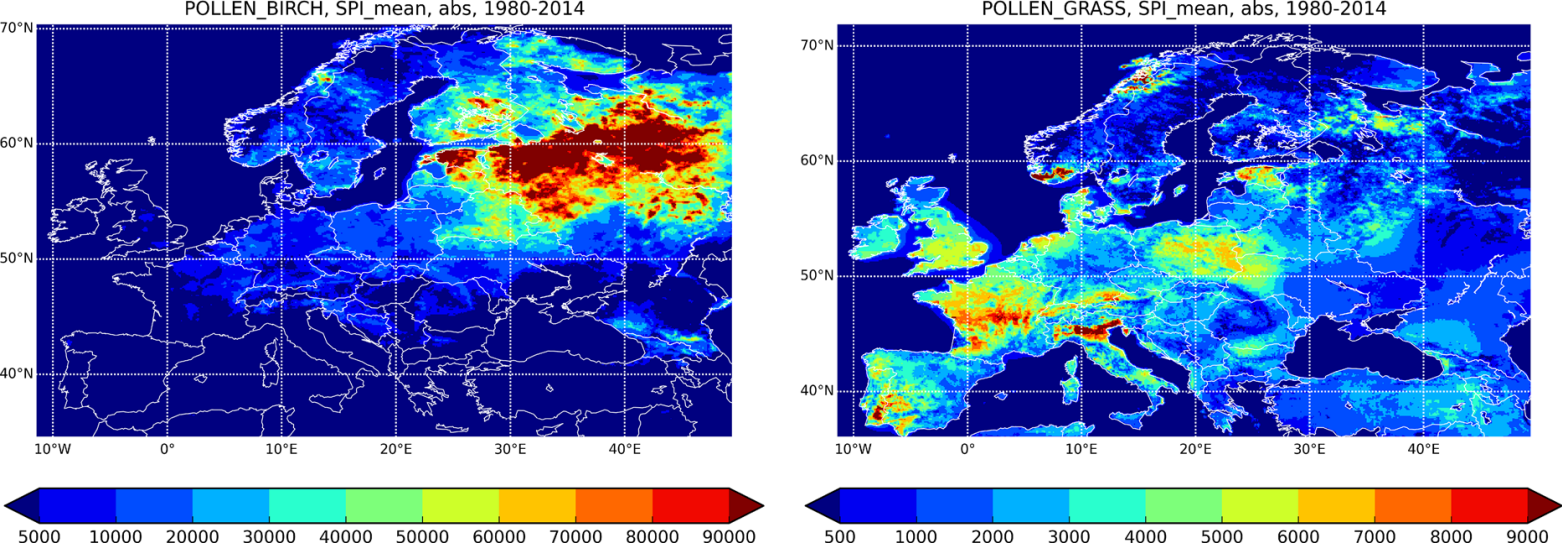
Mean SPI, 1980–2014 for birch (*left-hand panel*) and grass (*right-hand panel*). Unit: (day pollen m^−3^)

The mean SPI averaged over 35 years (Fig. 4, birch on the left-hand and grass on the right-hand panels) is expectedly concentrated over the areas with maximum pollen production (Fig. 1). For birch, these are mainly in North-Eastern Europe and Central Russia, with the rest of Europe experiencing 10–1000 times lower load. The grass SPI is distributed more homogeneously, showing high load in France, Po Valley, UK, Poland, Estonia, south of Iberian Peninsula, etc. The pattern for the grass SPI also has visibly sharper local features: it quickly follows the changes in the grass productivity map. This is a consequence of the shorter travelling distance of the grass pollen: these large particles are quickly removed from the atmosphere.

**Fig. 5:**
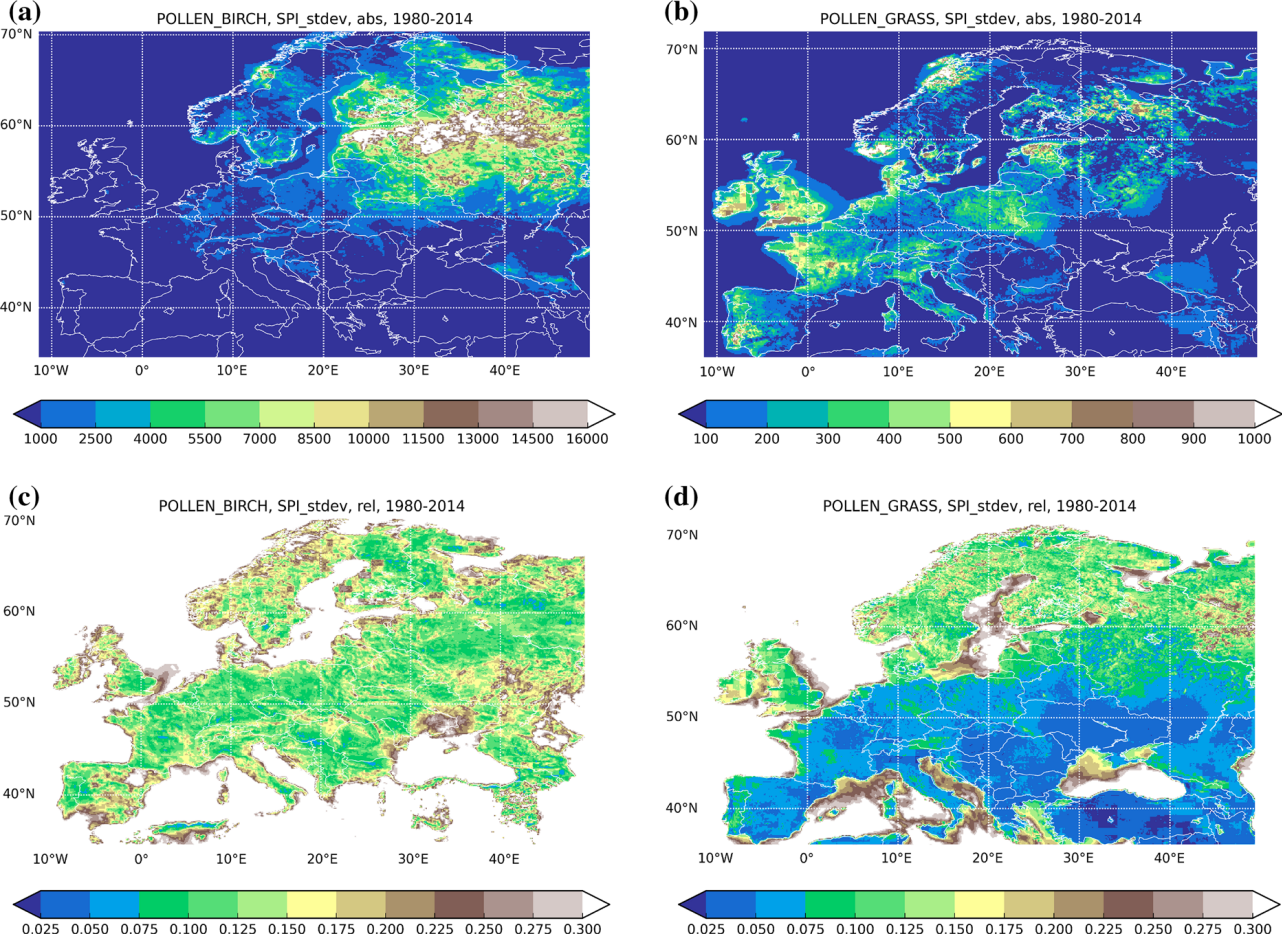
Absolute (*upper row*) and normalised with the mean SPI Fig. 4 (*lower row*) standard deviation of the SPI, 1980–2014. Species: birch (*left column*) and grass (*right column*). Unit: (day pollen m^−3^) for *upper row*, relative unit for *lower row*

**Fig. 6:**
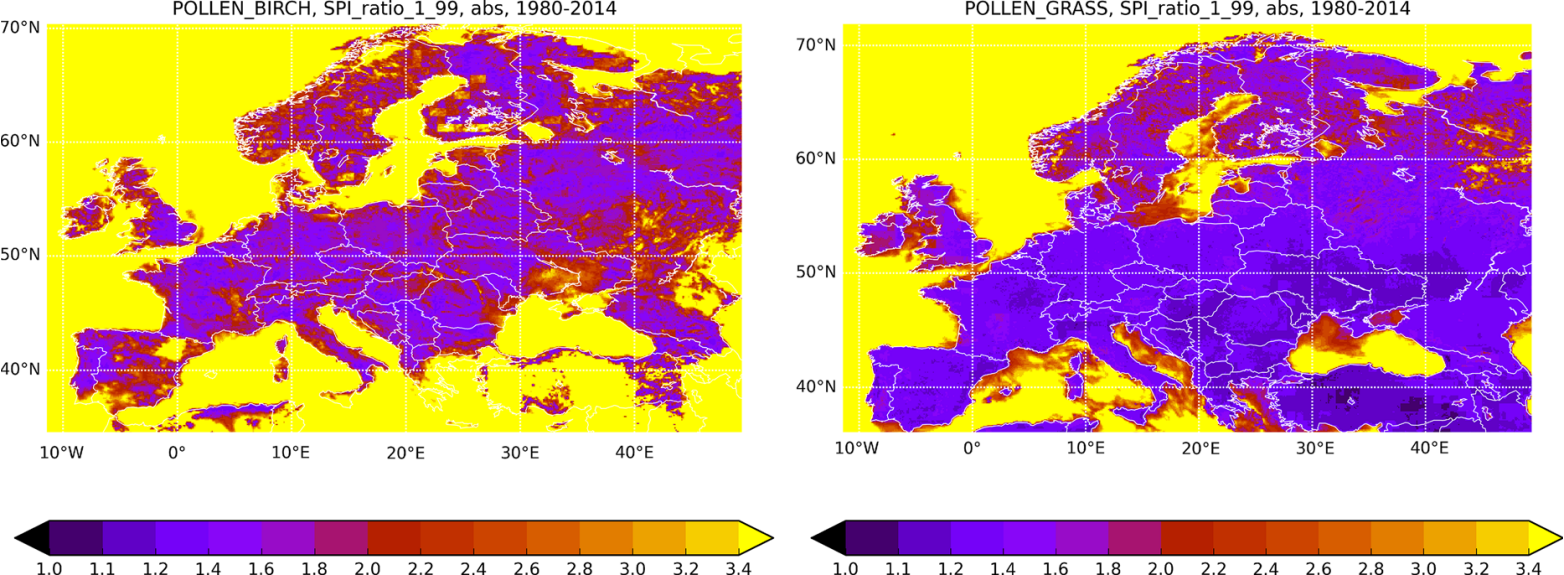
Range of the SPI variability: ratio max/min SPI during 1980–2014. Relative unit

The SPI variability is presented in two different ways: as the standard deviation (Fig. 5) and via the ratio of max and min SPI during the 35-year period (Fig. 6). Comparison of the SPI absolute and normalised (with the mean SPI, Fig. 4) standard deviations (Fig. 5) reveals several important features of the transport-induced variability.

For birch (Fig. 5, left column), absolute standard deviation (upper row) closely follows the mean SPI pattern of Fig. 4, though not directly proportional to it. For grass, the mean SPI and its standard deviation differ substantially. The SPI in coastal regions tends to vary more than inland (e.g. south-west of the UK, western coast of France, and south of Sweden). Conversely, high SPI in Po Valley seems to be very stable: its absolute deviation is comparable with the rest of Italy and Germany despite the several times higher mean SPI.

The relative standard deviation (*σ*) and the max/min ratio (Fig. 6) are surprisingly homogeneous over the whole European continent (with a few exceptions discussed further): for birch *σ*~15–25% and for grass *σ* ~5–15%. For grass, variability in the north is somewhat higher: the normalised standard deviation reaches 20%. For birch, it stays practically constant all over Europe, only slightly increasing in Scandinavia up to 20–25%.

## Discussion

### Peculiarities of the modelled variability patterns for birch and grass

For both species, the highest normalised SPI standard deviation (Fig. 5c, d) is over sea areas, where it is completely determined by the variability of transport from the continent. One can point out that in terrestrial regions, which are free from birches, the situation is similar, e.g. the steppes and the deserts east and north of Caspian Sea, which are too hot for birch, and the Russian tundra in the north, which is too cold. The model predicted just a few blows reaching these territories, which resulted in low mean SPI with high relative variability.

A few regions appeared to have intriguingly low or high variability. For instance, north-east of Spain showed practically the largest variability of birch among the terrestrial areas (Figs. 5c, 6 left). This area coincides with the regional minimum of the birch SPI [below 100 (pollen day m^−3^), Fig. 4] and essentially zero local pollen emission. In surroundings, however, birch is present (at least in Pyrenees). Therefore, a plausible explanation is that the regional SPI there, similarly to the sea areas and other birch-free regions, is dictated by the atmospheric transport. A similar situation is in south of Ukraine including Crimea Peninsula: birches in the north and north-west decide the regional SPI via irregular blows.

Surprising patterns are shown in the mountainous regions. The topographic specifics are not visible at all from the corresponding patterns: variability stays almost the same in the mountains and nearby flat-terrain regions. Out of common sense, one would expect mountains to be prone to strong jumps between the years due to quickly changing specific weather conditions, inhomogeneous distribution of vegetation and precipitation fields, delayed pollen season compare to nearby areas, etc. But this has not materialised in the simulations. One of the possible explanations is a coarse resolution of the meteorological data used for the reanalysis: 0.72° lon–lat. This is not enough to resolve the details of complex-terrain meteorology. The high resolution of the birch habitation map (1 km) and simulations (10 km) are more adequate but seemingly could not compensate the lack of topography-resolving meteorological fields. Therefore, the low transport-induced SPI variability in the mountains should be taken with care and verified with simulations driven by high-resolution meteorological data.

There is also no clear relation between the normalised variability and the source areas: even vast birch forests in Russia have not left any noticeable trace in Figs. 5c and 6, left. Considering this result together with the sharp rise of the standard deviation over the sea areas and birch-free regions, one has to accept that even comparatively low local pollen production practically decides both the mean SPI and its inter-annual variability: long-range transport has a limited impact on SPI if the local production exists. This observation leads to the main conclusion of the current analysis: the local environmental conditions controlling the near-surface pollen concentrations in the source areas are the main drivers of the transport-induced SPI variability in terrestrial regions. These are: ventilation driven by horizontal wind speed, vertical mixing driven by turbulence, uplift due to vertical wind component and convection, scavenging with precipitation, ambient humidity modulating the water uptake by pollens and therefore changing their sedimentation velocity.

The model showed systematic growth of the relative variability towards the north, both for grass and birch (Figs. 5, 6). It looks like the SPI is more sensitive to transport conditions in the north than in Central and Southern Europe. The elevated max/min ratio in Siberia suggests that the same is valid for strongly continental climatic conditions.

### How reliable are the obtained estimates?

The results presented in the previous section are obtained from the model simulations and, in essence, represent a sensitivity test for SILAM with regard to varying the meteorological conditions. As it was already mentioned, the effect is not directly observable since measurements cannot separate the meteorological, the biological and the external reasons for the SPI variability. Then, what is the relation of the obtained model sensitivity and real-world phenomena? To justify the results, one has to make sure that: (1) SILAM accounts for the major factors affecting the pollen dispersion and that the errors in the corresponding modules are small, (2) there are at least some indirect confirmations of the effect derived from observational data.

#### Reliability of the SILAM model and input data

SILAM is a heavily used and constantly evaluated chemical transport model, which incorporates all main transport-related processes.

Transport with wind and mixing are the major processes responsible for the pollen dispersion. Inside the model, they are represented by advection (Sofiev et al. 2015b) and diffusion (Sofiev 2002) mechanisms. As shown in those descriptions, their accuracy is high, so that one can neglect the contribution of the dispersion numerical errors to the SPI variability.

Pollen removal via dry deposition follows (Kouznetsov and Sofiev 2012), which derives the formulations starting from the basic principles and compares the obtained dependencies with numerous wind-tunnel and real-world data with high scores. Gravitational settling and inertia of pollen grains were examined by Sofiev et al. (2006), who confirmed applicability of the standard technique to these particles. Wet deposition for pollen is strongly dominated by the sub-cloud scavenging, i.e. the impaction mechanism is by far the most important. Its description and evaluation are also presented in Kouznetsov and Sofiev (2012).

The removal efficiency depends on the pollen size and density, which depend on water uptake by the grain—the process not included in the computations. As a result, variation of the mean humidity from year to year would affect the SPI variability estimates. However, Sofiev and Prank (2016) showed that the inter-annual variation of humidity is very small if averaged over time and a region. Therefore, omission of this phenomenon cannot significantly affect the results of this study (the related errors are of systematic character and affect all years in a similar way).

The most uncertain is evidently the pollen source term itself: the release description determines the sensitivity of the model to the meteorological conditions at the emission location. Evaluation of the birch source term (Siljamo et al. 2012; Sofiev et al. 2012b) showed that it includes all main processes: dependence on humidity, temperature, and wind. Uncertainties are large but integration over time towards SPI averages them out.

The grass source causes more concerns due to its fixed season timing. However, the missing variability is small compare to the length of the season: start is usually within 5 days from its mean date, which is just 5–10% of the typical season duration (Emberlin et al. 1999; Jochner et al. 2015; Kasprzyk 2006; Rodríguez-Rajo et al. 2009; Ribeiro et al. 2007; Sikoparija et al. 2006, etc.). This is in sharp contrast to birch, which timing can deviate for a week or two from the long-term average (Chmielewski and Rötzer 2002). But this variability is successfully captured by SILAM (Sofiev et al. 2015a; Siljamo et al. 2012; Sofiev et al. 2012b). Uncertainty of the season end is larger for both species—about 10 days—but also the concentrations get low. As a result, the contribution of the fixed grass season timing to the overall uncertainty is hardly more than 5–10%.

The uncertainties of the input meteorological fields and their interpretation by the SILAM preprocessor are the most difficult to quantify. The ERA-Interim reanalysis heavily uses data assimilation, which prevents its departure from the actual weather pattern, especially in Europe where the meteorological network is dense (Dee et al. 2011). However, even comparatively small perturbations in meteorology can result in substantial changes in boundary-layer characteristics (Sofiev et al. 2010). As a rough estimate, resulting uncertainty in the daily concentrations can be as large as a factor of 1.5. After averaging over 20 and 60 days of the typical birch and grass season duration, we obtain additional ~11 and 7% of uncertainty for birch and grass, respectively.

Summarising, internal errors and shortcuts of the model and input data roughly mount to about 15 and 10% of the SPI uncertainty for birch and grass, respectively.

#### Indirect observational evidence

Direct verification of the above modelling findings is hardly possible. Indeed, the pollen observations register the total variability of the SPI, which is due to changes in total annual pollen production, external forcing, and the current-season dispersion conditions. Their explicit separation is not straightforward.

Nearly the only possibility to indirectly evaluate the above analysis is to relate it with the measurement-based statistical models—those, which used both year-0 and year-1 meteorology to predict the SPI. Albeit few, such studies show quite clear picture (Emberlin et al. 2007; Latalova et al. 2002; Stach et al. 2008). In particular, four out of five locations (Poznan, Gdansk, Worcester, London, but not Krakow) showed significant correlations of the birch SPI with the year-1 meteorological conditions.

The year-1 parameters used in the multi-linear regression models by Latalova et al. (2002) and Stach et al. (2008) were: (1) several quantities describing the rain amounts during various 10-day or monthly periods (all ended up in the SPI-predicting equation with negative signs, i.e. reducing the SPI), (2) monthly mean (with positive sign) and max (with negative sign) temperature, and (3) mean wind speed, with negative sign. These parameters correspond to the processes mentioned in the previous section: item (1) describes the removal of pollen from the air, (2) and (3) refer to ventilation and turbulent mixing. The way these processes affect the SPI is also comparatively clear. Precipitations remove pollen from the air, thus very efficiently reducing the SPI. Maximum temperature is reached in anti-cyclonic conditions during hot sunny days with strong vertical convective mixing but low horizontal wind speed. These two processes compete but negative sign in the final regression model suggests that the net effect is negative: the near-surface concentrations get reduced by up-mixing the pollen clouds. Wind is also a characteristic of efficiency of ventilation, this time due to horizontal transport. A somewhat unclear mechanism is only behind the positive correlation with mean daily temperature: it indeed pushes up the daily emission but not the total amount of pollen, which is prescribed by the conditions of the previous year 0. Therefore, its season-wide impact should be zero at least in case of birch. One of the possible explanations is that in spring temperature is anti-correlated with precipitation and humidity, i.e. serves as another “dryness” characteristic associated with the low pollen removal.

Comparing the lists of processes identified from the model calculations and statistical observation-based models, one can see that they are very similar and both point at importance of the current year-1 meteorology over source areas. Most of these processes can efficiently reduce the SPI in the source vicinity (removal, ventilation, uplifting) but are much less powerful in increasing it in remote areas: transported pollen clouds get diluted in the course of transport simultaneously loosing pollen due to sedimentation.

### How large is the transport-induced SPI variability compare to the total?

As pointed out in the previous sections, the standard deviation of the SPI is a few tens of %, about twice more for birch than for grass. The max/min ratio (Fig. 6) reaches a factor of 2 for grass and exceeds 3.5 for birch, both higher in the north than in the south. Areas with no local pollen production show many times stronger variability.

The modelled transport-induced SPI variability can be compared with the total variability based on the observations and reported in the literature (Emberlin et al. 2007; Latalova et al. 2002; Stach et al. 2008; Rodríguez-Rajo et al. 2006; García de León et al. 2015, etc.).

Detailed analysis of the summaries presented in those papers would require going back to raw data, reprocessing them with a homogeneous definition of pollen season, treatment of missing data, accounting for different lengths of the time series, specifics of the station locations, etc. These are left outside this short paper. A rough but sufficiently conclusive comparison can be performed using the data already reported in those papers.

The comparison shows that for grass, the transport-induced variability is about 15% of the observed total one. For instance, the observed max–min ratio in Cordoba, Spain (García de León et al. 2015), is almost an order of magnitude, whereas transport brings about a factor of 1.5 or less (Fig. 6). In London, the SPI deviation from the 13-year average (1990–2002) is from −1000 pollen day m^−3^ up to 2000 pollen day m^−3^ (Smith and Emberlin 2005), i.e. the absolute max–min SPI difference is about 3000 pollen day m^−3^. The transport-induced range predicted here is ~800 pollen day m^−3^ at the Holloway (varies from 500 pollen day m^−3^ for Central London up to 1000 pollen day m^−3^ for suburbs), i.e. 4 times lower, despite three times longer modelled period.

For birch, the transport importance is higher than for grass, which is projected to the SPI variability: the transport contribution turned out to be about 20–40%. Thus, the observed total-variability max/min ratio of 20 was reported by Ranta et al. (2008) for several locations in Southern Finland (one outlying point removed). For the same region, current study reported the transport-induced max/min ratio of ~4, which constitutes 20% of the total variability. For Poland, the observed total max/min ratio was about 7 ((Stach et al. 2008), 11 years of observations), whereas the predicted transport-induced one is about 2.5, i.e. 30% of the total. The same work reported the birch SPI for London and Worcester, where the max/min ratio slightly exceeded 10 (somewhat higher variability in Worcester than in London). The transport-induced max/min ratio is 4 for London and 6 for Worcester, i.e. almost 50% of the total one.

## Conclusions

The results of the SILAM model reanalysis of birch and grass dispersion in Europe in 1980–2014 demonstrated a substantial contribution of dispersion conditions during the flowering season (year 1) to the modelled Seasonal Pollen Index SPI and, consequently, to the inter-annual variation of the modelled SPI. The same conclusion follows from (albeit, few) statistical models predicting the SPI using meteorology of both year 0 (previous year) and year 1.

The transport-induced SPI variability was quantified via sensitivity of SILAM-predicted SPI to the dispersion conditions. It was quite homogeneous over Europe and mounted to 15–20% for birch and 5–15% for grass, measured as the SPI standard deviation normalised by its multi-annual mean value. For grass, there is a tendency towards higher variability in the north, where the normalised standard deviation reaches 20%. Higher variability of the grass SPI was also observed over several coastal regions. For birch, the normalised variability stays practically constant all over Europe, only slightly increasing in Scandinavia, where it sometimes reaches 20–25%.

Uncertainty related to the modelling experiment set-up and SILAM errors mount to ~15% for birch and 10% for grass.

Computations suggested that the normalised standard deviation is not sensitive to local topography, but the resolution of input meteorology is too coarse for firm conclusions.

The variability is much higher in the regions with no local pollen production. It reflects the higher variability of the pollen long-range transport in comparison with the local dispersion and removal conditions, but also shows that already small local sources are important contributors to the SPI.

A quick comparison with the literature-reported observed total SPI variability showed that the transport-related contribution is comparatively small for grass—15–20%—but twice larger for birch—20–40%.
